# Supplementary material for: Early Diagnosis of Fibromyalgia Using Surface-Enhanced Raman Spectroscopy Combined with Chemometrics
Source: Biomedicines. 2024 Jan 9;12(1):133. doi: 10.3390/biomedicines12010133 (PMC10813180; doi:10.3390/biomedicines12010133)
Supplement: Supplementary file 1 [file biomedicines-12-00133-s001.zip › biomedicines-2812302-supplementary.pdf]

**Supplementary data**

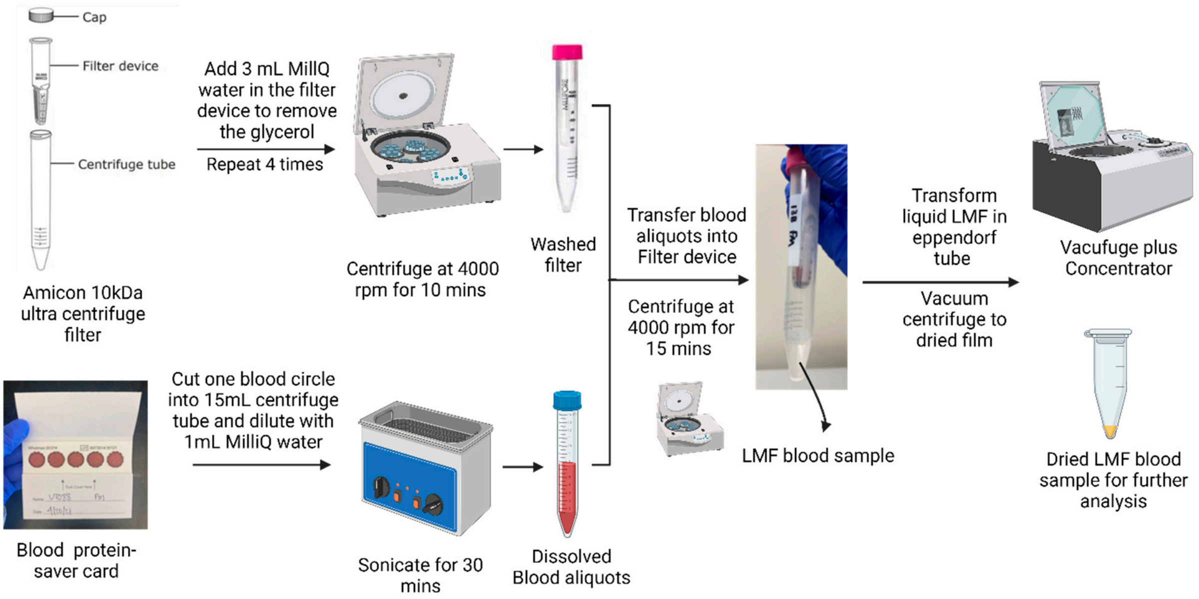


**Figure S1.** Extraction procedure of low-molecular weight fraction (LMF) of the human plasma proteome.

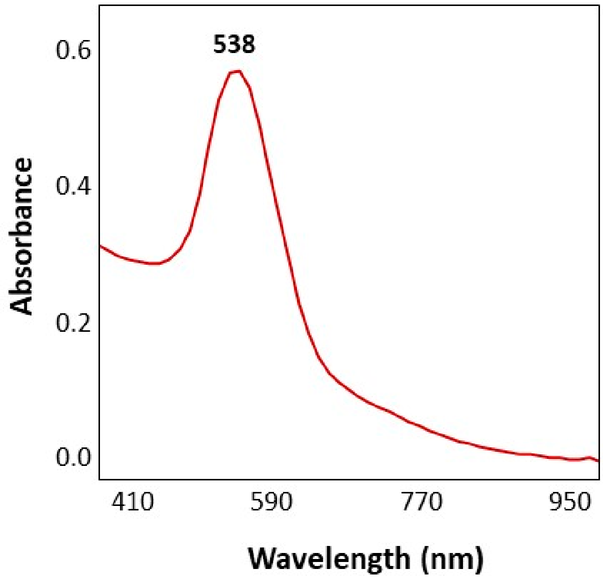


**Figure S2.** UV-vis spectra of AuNPs with maximum extinction at 538 nm.

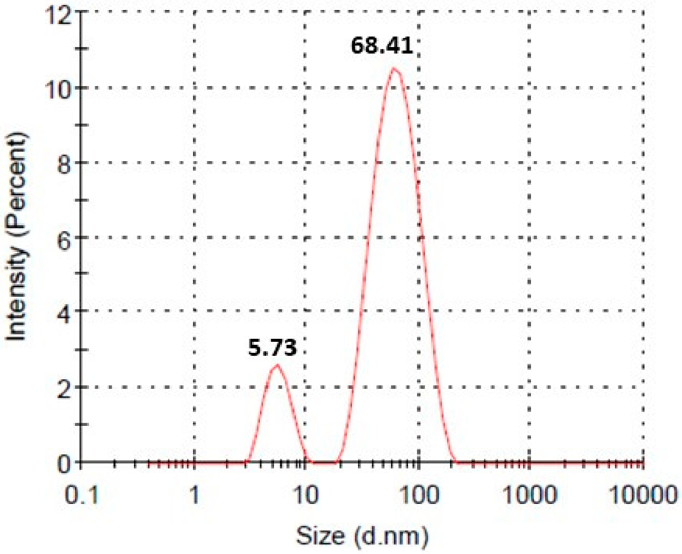


**Figure S3.** AuNPs size distribution by intensity.

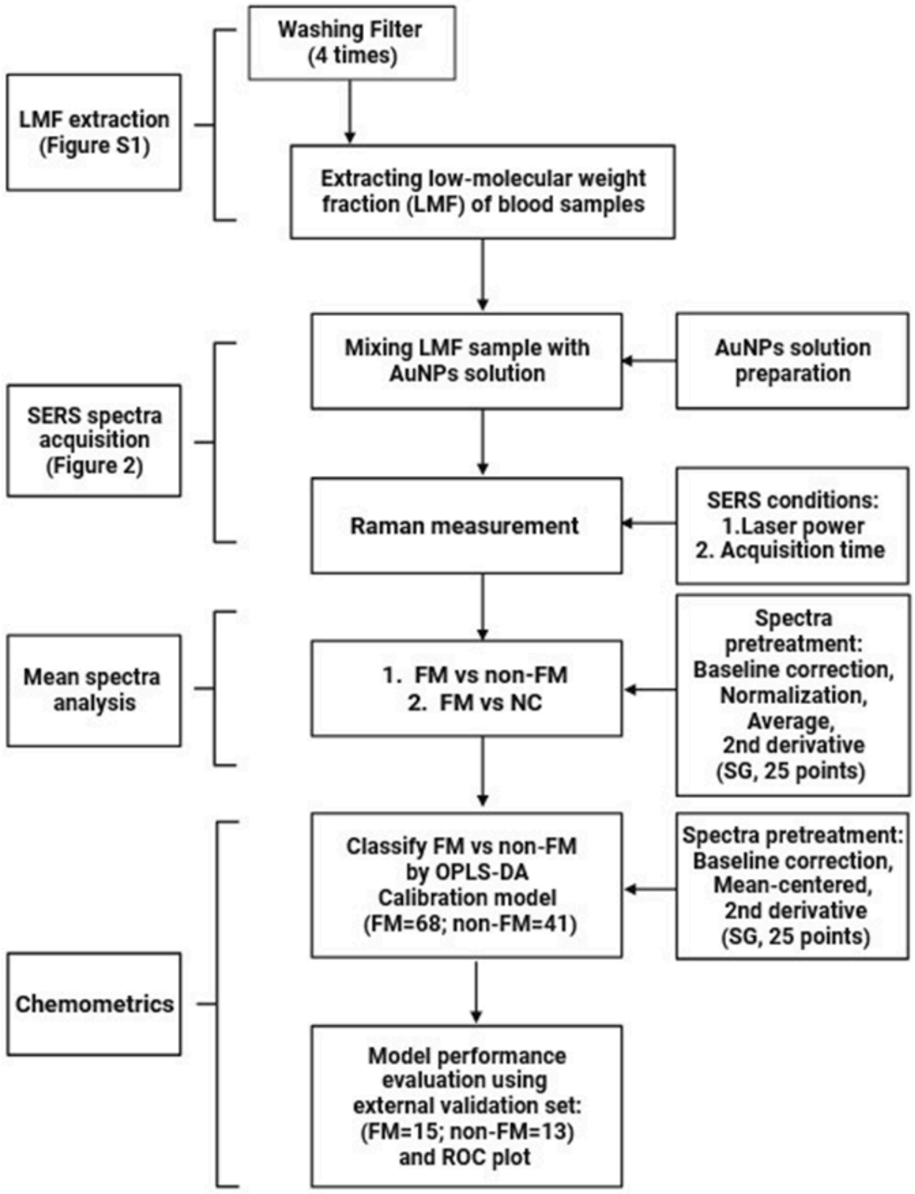


**Figure S4.** Flow diagram of the entire study process.
